# Greater risk of severe COVID-19 in Black, Asian and Minority Ethnic populations is not explained by cardiometabolic, socioeconomic or behavioural factors, or by 25(OH)-vitamin D status: study of 1326 cases from the UK Biobank

**DOI:** 10.1093/pubmed/fdaa095

**Published:** 2020-06-19

**Authors:** Zahra Raisi-Estabragh, Celeste McCracken, Mae S Bethell, Jackie Cooper, Cyrus Cooper, Mark J Caulfield, Patricia B Munroe, Nicholas C Harvey, Steffen E Petersen

**Affiliations:** 1 William Harvey Research Institute, NIHR Barts Biomedical Research Centre, Queen Mary University of London, London, UK; 2 Barts Heart Centre, St Bartholomew’s Hospital, Barts Health NHS Trust, London, UK; 3 North West Anglia NHS Foundation Trust, Hinchingbrooke Hospital, Huntingdon, UK; 4 MRC Lifecourse Epidemiology Unit, University of Southampton, Southampton, UK; 5 NIHR Southampton Biomedical Research Centre, University of Southampton and University Hospital Southampton NHS Foundation Trust, Southampton, UK; 6 NIHR Biomedical Research Centre, University of Oxford, Oxford, UK

**Keywords:** communicable diseases, epidemiology, public health

## Abstract

**Background:**

We examined whether the greater severity of coronavirus disease 2019 (COVID-19) amongst men and Black, Asian and Minority Ethnic (BAME) individuals is explained by cardiometabolic, socio-economic or behavioural factors.

**Methods:**

We studied 4510 UK Biobank participants tested for COVID-19 (positive, *n* = 1326). Multivariate logistic regression models including age, sex and ethnicity were used to test whether addition of (1) cardiometabolic factors [diabetes, hypertension, high cholesterol, prior myocardial infarction, smoking and body mass index (BMI)]; (2) 25(OH)-vitamin D; (3) poor diet; (4) Townsend deprivation score; (5) housing (home type, overcrowding) or (6) behavioural factors (sociability, risk taking) attenuated sex/ethnicity associations with COVID-19 status.

**Results:**

There was over-representation of men and BAME ethnicities in the COVID-19 positive group. BAME individuals had, on average, poorer cardiometabolic profile, lower 25(OH)-vitamin D, greater material deprivation, and were more likely to live in larger households and in flats/apartments. Male sex, BAME ethnicity, higher BMI, higher Townsend deprivation score and household overcrowding were independently associated with significantly greater odds of COVID-19. The pattern of association was consistent for men and women; cardiometabolic, socio-demographic and behavioural factors did not attenuate sex/ethnicity associations.

**Conclusions:**

In this study, sex and ethnicity differential pattern of COVID-19 was not adequately explained by variations in cardiometabolic factors, 25(OH)-vitamin D levels or socio-economic factors. Factors which underlie ethnic differences in COVID-19 may not be easily captured, and so investigation of alternative biological and genetic susceptibilities as well as more comprehensive assessment of the complex economic, social and behavioural differences should be prioritised.

## Introduction

The coronavirus disease 2019 (COVID-19) pandemic has to date resulted in over 6 million cases and 376 000 deaths worldwide^1^. Growing reports highlight men and Black, Asian and Minority Ethnic (BAME) cohorts as at higher risk of adverse COVID-19 outcomes^2^^,^^3^. Variations in cardiometabolic disease burden^4^, oestrogen pathway activity^5^, vitamin D levels^6^ and angiotensin-converting enzyme (ACE) 2 receptor expression^7^ have been proposed as potential explanations for the differential pattern of disease severity. Furthermore, disparities in socio-economic standards, housing conditions, socialization habits and risk perception have potential implications for risk of exposure and transmission. Understanding the significance of these factors is urgently needed to inform public health and research efforts.

We therefore investigated, in the UK Biobank (UKB) cohort, whether differential patterns of COVID-19 incidence and severity by sex and ethnicity might be explained by cardiometabolic, socio-economic, lifestyle and behavioural exposures.

## Methods

### Setting and study population

UKB is a prospective cohort study of over half a million men and women from across the UK covering a range of urban and rural settings. Recruitment was between 2006 and 2010 through postal invite of individuals aged 40–69 years old identified through National Health Service (NHS) registers. All individuals living within 10 miles of one of 22 UKB assessment centres were invited to participate. Individuals who were unable to consent were not recruited. Baseline assessment included detailed characterization of socio-demographics, lifestyle, health, a series of physical measures and blood biochemistry. The protocol is publicly available^8^. Data linkage with Hospital Episode Statistics (HES) enables prospective tracking of health outcomes for all participants with conditions recorded according to international classification of disease (ICD). Incidence of key events, such as myocardial infarction (MI), is algorithmically defined by cross-checking over multiple data sources^9^. Linkage with Public Health England has enabled rapid release of linked COVID-19 test results of UKB participants to researchers^10^. The latest data release (29 May 2020) included test results from 16 March 2020 to 18 May 2020. As UK testing during this period was almost entirely restricted to hospitalized patients, researchers have been advised that COVID-19 positive status can be taken as surrogate for severe disease^11^.

### Exposures

We considered relevant demographic (age, sex, ethnicity), biological (cardiometabolic, 25(OH)-vitamin D status), socio-economic (material deprivation, type of home, household overcrowding, poor diet quality) and behavioural (sociability, attitude to risk) exposures (Supplementary Table 1).

**Fig. 1:**
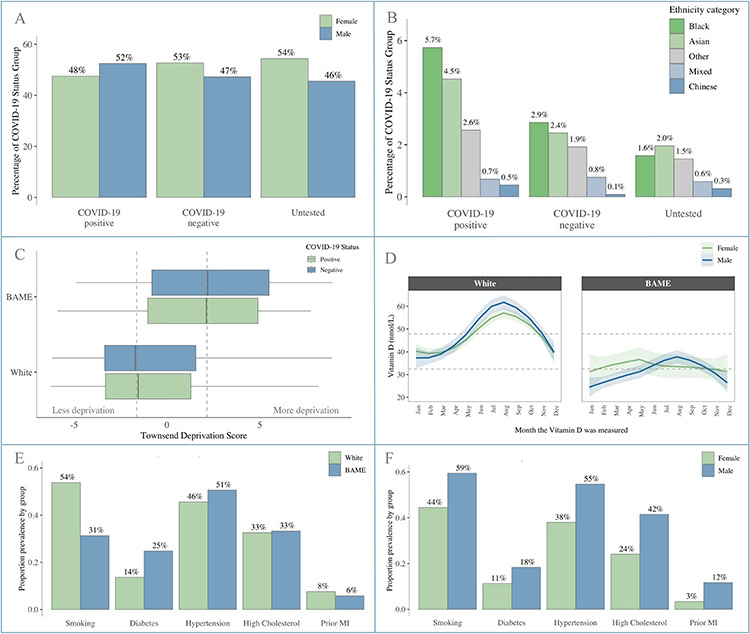
Baseline participant characteristics. Panel A: Male: Female split by COVID-19 status; Panel B: Percentage of participants from different BAME ethnicities by COVID-19 status; Panel C: Townsend deprivation score by ethnicity and COVID-19 status; Panel D: Vitamin D levels by month of measurement stratified by sex and ethnicity; Panel E: Cardiometabolic profile stratified by ethnicity; Panel F: Cardiometabolic profile stratified by sex.

We used age and sex as recorded at baseline. For consistency with wider UK classification, we document ethnicity as White and BAME. For the latter we report breakdown of ethnicities as per existing UKB categories: Black (Caribbean, African, any other Black background), Asian (Indian, Pakistani, Bangladeshi, any other Asian background), Chinese, Mixed (White and Black Caribbean, White and Black African, White and Asian, any other mixed background) and ‘other’. Townsend deprivation score is reported by the UKB as a measure of material deprivation calculated at baseline: zero, positive and negative scores correspond to average, higher and lower levels of deprivation, respectively, relative to national averages^12^. We used type of housing as a binary variable comprising communal living spaces (flat, apartment, sheltered accommodation) versus stand-alone housing (house, bungalow). We considered household overcrowding based on self-report of household size and intergenerational cohabitation. Socialization habits were defined per self-reports of frequency of family/friend visits and participation in regular leisure activities outside the home. Attitude to risk was assessed using self-report of tendency ‘to take risks’. Body mass index (BMI) was calculated from height and weight recorded at baseline. Smoking status was based on self-report. Hypertension, diabetes and hypercholesterolaemia were defined through cross-checking across self-report and HES data. A list of ICD codes used is available in Supplementary Table 2. Prior MI was obtained from UKB algorithmically defined health outcomes. We used serum 25(OH)-vitamin D levels measured at baseline [Clinical Laboratory Improvement Amendments (CLIA) analysis on a DiaSorin Ltd. LIASON XL], limiting to results between 10 and 375 nmol/L based on the manufacturer’s analytic range^13^. We adjusted for seasonality by regressing vitamin D on month of sampling as a factor; this allowed derivation of vitamin D adjusted to the same month for each participant. There were differences in vitamin D levels and degree of seasonal variation by ethnicity (Fig. 1D). We therefore performed seasonality adjustment separately for White and BAME populations and added the intercept to the adjusted variables to maintain the difference between the two groups. We considered processed meat intake as a marker of poor diet quality. We converted self-reported weekly intake frequencies into probabilities of daily intake and multiplied by portion size to derive a continuous measure of daily consumption in grams, as previously published using this dataset^14^^,^^15^.

### Ethics

This study was covered by the ethics approval for UKB studies from the NHS National Research Ethics Service on 17 June 2011 (Ref 11/NW/0382) and extended on 10 May 2016 (Ref 16/NW/0274).

### Statistical analysis

Statistical analysis was performed using R Version 3.6.2 [R Core Team (2019). R: A language and environment for statistical computing. R Foundation for Statistical Computing, Vienna, Austria. URL https://www.R-project.org/] and RStudio Version 1.2.5019 [RStudio Team (2015). RStudio: Integrated Development for R. RStudio, Inc., Boston, MA URL http://www.rstudio.com/].

UKB participants were grouped according to COVID-19 status: test positive, test negative and untested. In the analysis of an earlier data release, we demonstrated similar associations when comparing the untested cohort with both the test negatives and test positives, suggesting that comparison with the whole cohort reveals associations with general hospitalization rather than specifically with COVID-19^16^. Therefore, to avoid bias relating to hospitalization, in the present study, we limited to modelling within the tested cohort. We performed analyses in the whole tested sample, and separately in men and women. Logistic regression models were first used to examine univariate associations. We then undertook individual multivariate models for each hypothesis to minimize loss of participants due to missingness from adding multiple variables simultaneously. We defined a final model using variables noted to be important from previous model permutations. We tested for multicollinearity setting a variance inflation factor (VIF) cut-off of 2.5. We present odds ratio (OR) for each exposure with the corresponding 95% confidence interval (CI) and *P*-value.

## RESULTS

### Population characteristics

#### Sex and ethnicity

Test results for 4510 participants were available (positive, *n* = 1326; negative, *n* = 3184). Baseline characteristics are summarized in Table 1. Comparisons with the untested cohort (*n* = 497 996) and characteristics by sex and ethnicity are summarized in Supplementary Tables 3, 4 and 5. There was over-representation of men and BAME ethnicities in the test positive cohort (Fig. 1A and B). Individuals of Black and Asian ethnicity were most disproportionately affected with Black ethnicities contributing over 3.5× the number of positive cases than their representation in the untested cohort (Supplementary Table 3, Fig. 1B).

**Table 1:** Baseline demographics by COVID-19 status

	Test positive (*n* = 1326)	Test negative (*n* = 3184)
Men	696 (52.5%)	1,505 (47.3%)
Age	68.11 (± 9.23)	68.91 (± 8.72)
White ethnicity	1,141 (86.0%)	2,927 (91.9%)
BAME ethnicities (total)	174 (13.1%)	241 (7.6%)
Black ethnicity	76 (5.7%)	91 (2.9%)
Asian ethnicity	60 (4.5%)	78 (2.4%)
Chinese ethnicity	6 (0.5%)	3 (0.1%)
Mixed ethnicity	9 (0.7%)	24 (0.8%)
Other ethnicity*	34 (2.6%)	61 (1.9%)
Smoking (current or previous)	683 (51.5%)	1,653 (51.9%)
Processed meat intake (g/day)	17.08 (± 15.67)	16.33 (± 15.00)
BMI (kg/m^2^)	28.04 [± 6.47]	27.41 [± 6.37]
Diabetes	217 (16.4%)	449 (14.1%)
Hypertension	624 (47.1%)	1,457 (45.8%)
High cholesterol	437 (33.0%)	1,034 (32.5%)
Prior MI	96 (7.2%)	242 (7.6%)
Vitamin D (nmol/L)**	33.88 [± 27.01]	35.45 [± 26.78]
Townsend deprivation score	−0.91 [± 5.34]	−1.55 [± 5.00]
Home type (flat/apartment)	191 (14.4%)	455 (14.3%)
Household size	2.50 (± 1.31)	2.32 (± 1.22)
Number of generations in household	1.41 (± 0.52)	1.35 (± 0.50)
Family/friend visits	975 (73.5%)	2,438 (76.6%)
Regular leisure activity	897 (67.6%)	2,124 (66.7%)
Tendency to take risks	404 (30.5%)	916 (28.8%)

Results are number (percentage) for categorical and mean (standard deviation) or median [interquartile range] for continuous variables.

^*^Ethnicity was missing for *n* = 11 test positive and *n* = 16 test negative participants, these participants are included as part of ‘other ethnicity’ in this table but have been excluded from subsequent modelling.

^**^Vitamin D has been adjusted for seasonality.

#### Cardiometabolic factors and vitamin D

Men and BAME ethnicities had overall greater burden of cardiometabolic morbidities compared to women and White cohorts, respectively (Fig. 1E and F). Serum 25(OH)-vitamin D levels were, on average, higher in White ethnicities than BAME cohorts (Fig. 1D).

#### Socio-demographic and behavioural factors

In comparison to the test negatives, those with a positive test had greater levels of material deprivation and were more likely to live in crowded households (Fig. 1C). BAME individuals had, on average, higher levels of material deprivation by Townsend score compared to those of White ethnicity (Supplementary Table 4). The frequency of family/friend visits and leisure activities outside the home was similar between the test positive and test negative groups. There was greater tendency to risk-taking behaviour in the test positive cohort, which was greater in men versus women and in BAME versus White ethnicities.

**Table 2:** Multivariate logistic regression models testing the role of cardiometabolic factors (Model 1), vitamin D (Model 2) and poor diet (Model 3) in determining risk of COVID-19

	Exposures	Whole tested sample *n =* 4510	Men *n =* 2201	Women *n =* 2309
Model 1: sex, age, ethnicity, smoking, BMI, diabetes, hypertension, high cholesterol, prior MI	Male sex	1.28* [1.12, 1.46]	–	–
		4.05$\times$10^−4^	–	–
	Age	0.99* [0.98, 1.00]	1.00 [0.98, 1.01]	0.99* [0.97, 1.00]
		0.0157	0.5128	0.0097
	BAME ethnicity	1.78* [1.43, 2.20]	2.07* [1.50, 2.84]	1.55* [1.15, 2.09]
		1.88$\times$10^−7^	7.90$\times$10^−6^	0.0040
	Smoking (previous/current)	1.02 [0.89, 1.16]	1.12 [0.92, 1.36]	0.91 [0.75, 1.10]
		0.7961	0.2533	0.3352
	BMI (kg/m^2^)	1.02* [1.01, 1.03]	1.03* [1.01, 1.05]	1.02 [1.00, 1.03]
		0.0015	0.0051	0.0537
	Diabetes	1.08 [0.88, 1.32]	1.06 [0.82, 1.38]	1.08 [0.77, 1.49]
		0.4781	0.6529	0.6665
	Hypertension	1.01 [0.86, 1.18]	0.93 [0.74, 1.16]	1.11 [0.89, 1.40]
		0.8875	0.5004	0.3563
	High cholesterol	0.97 [0.82, 1.15]	1.04 [0.83, 1.31]	0.89 [0.68, 1.15]
		0.7479	0.7108	0.3690
	Prior MI	0.89 [0.68, 1.16]	0.85 [0.62, 1.15]	0.97 [0.55, 1.65]
		0.4041	0.2961	0.8990
Model 2: sex, age, ethnicity, vitamin D	Male sex	1.31* [1.14, 1.50]	–	–
		1.85$\times$10^−4^	–	–
	Age	0.99* [0.98, 1.00]	1.00 [0.99, 1.01]	0.99* [0.97, 1.00]
		0.0166	0.5500	0.0073
	BAME ethnicity	1.77* [1.41, 2.22]	2.02* [1.45, 2.82]	1.60* [1.16, 2.18]
		9.27$\times$10^−7^	3.51$\times$10^−5^	0.0038
	Vitamin D	1.00 [1.00, 1.00]	1.00 [1.00, 1.01]	1.00 [1.00, 1.01]
		0.7185	0.7464	0.9288
Model 3: sex, age, ethnicity, processed meat	Male sex	1.26* [1.10, 1.44]		
		8.55$\times$10^−4^		
	Age	0.99* [0.98, 1.00]	1.00 [0.99, 1.01]	0.99* [0.98, 1.00]
		0.0144	0.4993	0.0082
	BAME ethnicity	1.81* [1.46, 2.24]	2.08* [1.52, 2.85]	1.62* [1.21, 2.17]
		4.18$\times$10^−8^	4.95$\times$10^−6^	0.0011
	Processed meat intake (100 grams/day)	1.26 [0.81, 1.94]	1.01 [0.57, 1.77]	1.83 [0.91, 3.66]
		0.3032	0.9742	0.0871

Results are ORs, 95% CI and *P*-values for each exposure from three separate models (1, 2 and 3). Exposures are mutually adjusted.

**Table 3:** Multivariate logistic regression models testing the role of material deprivation (Model 4), housing conditions (Model 5) and final model (Model 6) in determining risk of COVID-19

	Exposures	Whole tested sample *n =* 4510	Men *n =* 2201	Women *n =* 2309
Model 4: sex, age, ethnicity, Townsend deprivation score	Male sex	1.27* [1.11, 1.45]	–	–
		3.87$\times$10^−4^	–	–
	Age	0.99* [0.98, 1.00]	1.00 [0.99, 1.01]	0.99* [0.98, 1.00]
		0.0222	0.6323	0.0089
	BAME ethnicity	1.67* [1.34, 2.07]	1.92* [1.39, 2.64]	1.49* [1.11, 2.01]
		3.94$\times$10^−6^	6.15$\times$10^−5^	0.0084
	Townsend deprivation score	1.03* [1.01, 1.05]	1.03* [1.00, 1.06]	1.03* [1.00, 1.06]
		0.0024	0.0402	0.0232
Model 5: sex, age, ethnicity, home type, household size^*^	Male sex	1.24* [1.09, 1.42]	–	–
		0.0016	–	–
	Age	1.00 [0.99, 1.01]	1.00 [0.99, 1.01]	0.99 [0.98, 1.00]
		0.3827	0.8207	0.1655
	BAME ethnicity	1.73* [1.39, 2.17]	1.86* [1.33, 2.59]	1.66* [1.22, 2.24]
		1.36$\times$10^−6^	2.60$\times$10^−4^	0.0011
	Home type	0.98 [0.80, 1.20]	1.05 [0.80, 1.38]	0.90 [0.66, 1.22]
		0.8650	0.7044	0.4918
	Household size	1.08* [1.02, 1.14]	1.08 [0.99, 1.18]	1.07 [0.99, 1.16]
		0.0140	0.0764	0.0941
Model 6 ‘final model’: sex, age, ethnicity, BMI, Townsend deprivation score, household size	Male sex	1.23* [1.08, 1.41]	–	–
		0.0021	–	–
	Age	1.00 [0.99, 1.00]	1.00 [0.99, 1.01]	0.99 [0.98, 1.00]
		0.3648	0.8297	0.1674
	BAME ethnicity	1.59* [1.26, 1.99]	1.74* [1.24, 2.45]	1.50* [1.10, 2.04]
		7.85$\times$10^−5^	0.0015	0.0105
	BMI (kg/m^2^)	1.02* [1.01, 1.03]	1.03* [1.01, 1.05]	1.02* [1.00, 1.03]
		9.71$\times$10^−4^	0.0036	0.0476
	Townsend deprivation score	1.03* [1.01, 1.06]	1.04* [1.01, 1.07]	1.03* [1.00, 1.06]
		0.0011	0.0133	0.0319
	Household size	1.09* [1.03, 1.16]	1.09 [1.00, 1.18]	1.10* [1.01, 1.19]
		0.0022	0.0529	0.0203

Results are ORs, 95% CI and *P*-values for each exposure from three separate models (4, 5 and 6). Exposures are mutually adjusted.

^*^Initial analyses additionally included number of generations in household, however, we observed significant multicollinearity between this variable and household size with higher VIF in the latter, hence it was removed from the final model.

### Univariate associations of exposures with COVID-19 positive status

We tested the univariate association of all defined exposures with COVID-19 positive status within the tested cohort (Supplementary Table 6). Male sex, BAME ethnicity, higher BMI, greater material deprivation and greater household overcrowding (household size, generations in household) were associated with increased odds of COVID-19 positive test. More frequent visits from family/friends were associated with lower risk of COVID-19 hospitalization, perhaps reflecting the role of social support in enabling individuals to remain at home when ill (given that a positive test implied hospital attendance). There was a negative association between age and COVID-19 positivity, which may reflect the narrow range and distribution of ages in the sample. Testing separately in men, BAME ethnicity, greater material deprivation and higher BMI were the only statistically significant exposures. For women, additionally, lower 25(OH)-vitamin D status, greater household overcrowding (household size, generations in household) and greater risk-taking behaviour were associated with COVID-19 positivity.

### Independent associations of specific exposures with COVID-19 status

#### Cardiometabolic factors

We undertook multivariate logistic regression models incorporating sex, age, ethnicity, smoking, BMI, diabetes, hypertension, high cholesterol and prior MI (Table 2, Model 1). Male sex and BAME ethnicity were associated with greater odds of COVID-19 positive status with OR 1.28 (1.12, 1.46) and 1.78 (1.43, 2.20), respectively. Every 1 kg/m^2^ of BMI was associated with 1.03 (1.01, 1.04) greater odds of COVID-19 positivity. There was a borderline negative association with age 0.99 [0.98, 1.00], which remained significant for women in sex-stratified analysis. There was no evidence of attenuation (compared with the crude models) in the associations with BAME ethnicity and higher BMI, consistent across men and women.

#### 25(OH)-vitamin D status and poor diet quality

In multivariate logistic regression models incorporating sex, age and ethnicity, there was no significant association between season-adjusted 25(OH)-vitamin D status and COVID-19 positivity (Table 2, Model 2). Similarly, in a separate model, adjustment for sex, age and ethnicity demonstrated no statistically significant association between processed meat consumption and COVID-19 status (Table 2, Model 3). In both models, male sex and BAME ethnicity were associated with higher odds of COVID-19 positive test across men and women, with no evidence of attenuation.

#### Material deprivation

We tested the effect of material deprivation in multivariate models with mutual adjustment for sex, age and ethnicity (Table 3, Model 4). There was a small, but statistically significant association between greater material deprivation and higher odds of COVID-19 positivity [OR 1.03 (1.01, 1.05)]. There remained strong and significant associations with male sex [OR 1.27 (1.11, 1.45)] and BAME ethnicity [OR 1.67 (1.34, 2.07)].

#### Housing conditions

We considered the effect of housing conditions in multivariate logistic regression models including sex, age, ethnicity, home type and household size. In the whole sample, male sex, BAME ethnicity and greater household size were associated with greater odds of COVID-19 positivity (Table 3, Model 5). Testing separately in men and women, BAME ethnicity was the only exposure which remained significantly associated with COVID-19 status. Attenuation of associations with household size is likely due to the small effect size and limited heterogeneity of the exposure in each of the sexes individually.

#### Socialization habits and attitudes to risk

We undertook separate multivariate logistic regression models testing for associations between COVID-19 status, socialization habits and risk-taking attitude (Supplementary Table 7) while adjusting for age, sex and ethnicity. Statistically significant associations were observed with male sex and BAME ethnicity which were not attenuated from crude models by adjustment for socialization or risk-taking attitude, which did not show significant associations.

### Final model

We built a final multivariate logistic regression model, with covariates selected based on previous model permutations including sex, age, ethnicity, BMI, Townsend score and household size (Table 3, Model 6). Male sex and BAME ethnicity were associated with greater odds of COVID-19 positivity: OR 1.23 (1.08, 1.41) and 1.59 (1.26, 1.99), respectively. Every 1 kg/m^2^ increase in BMI was associated with 1.02 (1.01, 1.03) greater odds of COVID-19 positivity and for every additional person living in the same household the odds increased by 1.09 (1.03, 1.16).

## Discussion

### Main finding of this study

In 4510 UKB participants tested for COVID-19 in a hospital setting, male sex, BAME ethnicity, higher BMI and greater household size were associated with significantly greater odds of a positive result. Despite variation in burden of cardiometabolic morbidities, 25(OH)-vitamin D levels and material deprivation by sex and ethnicity, these factors were not significantly associated with COVID-19 positivity and did not explain the strong association with ethnicity. The pattern of associations did not vary between men and women.

### What is already known on this topic

Mounting evidence suggests disproportionate adverse effects of COVID-19 in BAME populations^2^. UK national audit data demonstrate that up to one-third of COVID-19 patients requiring intensive care are from BAME backgrounds, a rate far greater than their representation in the general population^17^. An analysis of COVID-19 deaths amongst NHS staff, found that 64% of deaths were in BAME cohorts, markedly disproportionate to their 20% contribution to the NHS workforce^18^. The latest report from the Office of National Statistics (ONS) also demonstrates greater risk of COVID-19 mortality in BAME groups^19^; individuals of Black ethnicity had over 3.5× greater risk of COVID-19 death compared to Whites, followed by Asian ethnicities^19^. Similarly, in the USA, there has been growing concern over the disproportionate number of COVID-19 deaths amongst African Americans^20^. These patterns are echoed across Europe, with Nordic countries reporting as much as 10× greater risk of COVID-19 in Somali populations^21^. We had previously documented this preponderance of cases amongst BAME individuals in our analysis of the initial UKB data release^16^; here, we have confirmed the observation in this larger dataset, and importantly demonstrated a non-uniform impact across different BAME groups with highest rates amongst Black followed by Asian ethnicities.

The greater cardiometabolic burden in both BAME and male cohorts has been proposed as potentially important in driving adverse COVID-19 outcomes. In our analysis, cardiometabolic morbidities were not significantly associated with COVID-19 status in multivariate models and did not attenuate sex and ethnicity associations. This suggests that the greater cardiometabolic burden in BAME individuals does not account for the adverse COVID-19 outcomes in this group.

Consistent with our findings, data from the UK and the USA highlight obesity as a marker of poor COVID-19 outcomes, such as requirement for intensive care^22^. There are suggestions of a possible pathophysiological link between adiposity and COVID-19 severity. Wide expression of ACE2 receptors within adipose tissue is thought to promote binding and cellular entry of severe acute respiratory syndrome coronavirus 2 (SARS-CoV-2)^23^. It has been suggested that adipose tissue may act as a ‘viral reservoir’ thereby contributing to a more prolonged and severe illness^23^. In addition, adipose tissue is a known source of inflammatory cytokines, such as Interleukin 6^24^. This is hypothesized to be linked to the association of adiposity with greater likelihood of cytokine storms and the consequent risk of severe respiratory complications in COVID-19. Indeed, studies have demonstrated association of higher Interleukin 6 levels with respiratory failure and requirement for mechanical ventilation in COVID-19 patients^25^. Greater adiposity, as well as BAME ethnicity, is associated with lower 25(OH)-vitamin D status. Although the active 1,25(OH)_2_-vitamin D form has immune system functions^26^, evidence linking low 25(OH)-vitamin D [the circulating storage form, and poorly correlated with 1,25(OH)_2_-vitamin D] with COVID-19 disease have been contradictory^27^. In our study, we found no independent associations between 25(OH)-vitamin D status and COVID-19 disease, suggesting that the relationship is confounded by ethnicity and BMI. Interestingly, the BMI association was retained in multivariate models, suggesting a possible independent role for adiposity, which clearly deserves further investigation.

Socio-economic deprivation is associated with poorer global health outcomes^28^. It has been suggested that ethnic differences in COVID-19 severity may relate to clustering of material deprivation with BAME status^29^. In the UKB, material deprivation is reported using the Townsend score, which is based on four factors—employment, car ownership, home ownership and household overcrowding. Consistent with national reports, we found higher material deprivation in BAME individuals participants. In multivariate models including age, sex, ethnicity and Townsend score, there were significantly greater odds of COVID-19 with greater material deprivation, while the association with ethnicity appeared strong and significant. Testing separately for the effect of household overcrowding, this exposure appeared significant independent of sex, ethnicity, age and home type. This suggests that it may not be global economic deprivation, but specific aspects relating to household overcrowding that has relevance to COVID-19. Consistent with these observations, a survey of COVID-19 cases from New York reports the highest number of cases occurring in areas with the largest average household size^30^. Furthermore, analysis of UK cases by the ONS also demonstrates that material deprivation does not adequately explain the ethnic disparities in COVID-19 outcomes^19^.

Behavioural factors, in particular attitudes that may compromise adherence to lockdown measures, have been proposed as potentially important in determining risk of exposure to SARS-CoV-2^31^^,^^32^. In our analysis, we did not find socialization habits and attitude to risk to be significantly important in conferring COVID-19 positive status.

### What this study adds

This study is consistent with growing reports of higher risk of severe COVID-19 in men and BAME populations. The augmented risk in BAME populations is non-uniform and disproportionately affects Black and Asian ethnicities. Higher BMI, greater material deprivation and household overcrowding are independent risk factors for COVID-19. The sex and ethnicity differential pattern of COVID-19 is not adequately explained by variations in cardiometabolic factors, 25(OH)-vitamin D levels, socio-economic or behavioural factors. However, factors which underlie ethnic differences in COVID-19 may not be easily captured. Investigation of alternative biological and genetic susceptibilities as well as more comprehensive assessment of the complex economic, social and behavioural differences is warranted.

### Limitations of this study

Given the observational nature of the study, we cannot discern causal relationships, and although we controlled for a wide range of covariates, the possibility of residual confounding should be considered. The vitamin D levels used in this analysis are based on measurements taken at the UKB baseline visit; therefore, we cannot account for possible changes that may have occurred since this measurement was taken. However, there is evidence that vitamin D status tends to track with time, particularly after adjustment for season of blood draw^33^^,^^34^ (as we present in the current paper) and there is no reason to expect population level shifts in vitamin D levels in this time period. Studies with more recent vitamin D measures would be of interest. The limited age range in this dataset precludes widely generalizable conclusions about the effects of age, and there are clearly wider social, economic and behavioural factors beyond those which we were able to study in UK Biobank. Occupational factors may have relevance in determining risk of exposure and viral transmission; this topic requires detailed dedicated study. Aggregating all BAME populations may overlook important differences between ethnicities; studies in samples with greater ethnic diversity are needed.

## Supplementary Material

Supplementary_Table_1_fdaa095

Supplementary_Table_2_fdaa095

Supplementary_Table_3_fdaa095

Supplementary_Table_4_fdaa095

Supplementary_Table_5_fdaa095

Supplementary_Table_6_fdaa095

Supplementary_Table_7_fdaa095

## Notes

**Zahra Raisi-Estabragh,** Dr. Cardiology registrar and BHF clinical research fellow

**Celeste McCracken,** Ms. Statistician and data scientist

**Mae S. Bethell,** Dr. junior doctor (Foundation programme)

**Jackie Cooper,** Ms. Senior Statistician

**Cyrus Cooper,** Director, Professor

**Mark J. Caulfield,** Prof/Sir Co-Director

**Patricia B. Munroe,** Professor

**Nicholas C. Harvey,** Professor

**Steffen E. Petersen,** Professor
