# Supplementary material for: Greater risk of severe COVID-19 in Black, Asian and Minority Ethnic populations is not explained by cardiometabolic, socioeconomic or behavioural factors, or by 25(OH)-vitamin D status: study of 1326 cases from the UK Biobank
Source: J Public Health (Oxf). 2020 Jun 19;42(3):451–60. doi: 10.1093/pubmed/fdaa095 (PMC7449237; doi:10.1093/pubmed/fdaa095)
Supplement: Supplementary_Table_1_fdaa095 [file supplementary_table_1_fdaa095.docx]

**Supplementary Table 1. Exposures considered and their definitions in the present study**

| Exposure | Description | Data type |
| --- | --- | --- |
| Sex | Sex recorded at baseline | Binary categorical |
| Age | Age calculated as of 1^st^ April 2020 | Numerical continuous |
| Ethnicity | As recorded at baseline visit, per existing UKB categories: White, Black, Asian, Chinese, Mixed, Other | Categorical |
| Smoking | Self-report of current, previous, never- considered as: current/previous vs never | Binary categorical |
| BMI | Calculated from height and weight at baseline:  BMI = weight (Kg) /height(m)^2^ | Numerical continuous |
| Vitamin D | Serum levels measured at baseline visit; corrected for seasonality | Numerical continuous |
| Processed meat | Self-report of weekly intake frequency, converted to grams/day intake based on 1 portion=75g | Numerical continuous |
| Diabetes | Self-report and HES (Supplementary Table 2) | Binary categorical |
| Hypertension | Self-report and HES (Supplementary Table 2) | Binary categorical |
| High cholesterol | Self-report and HES (Supplementary Table 2) | Binary categorical |
| Prior MI | Algorithmically defined outcomes | Binary categorical |
| Townsend deprivation score | Score of relative material deprivation from baseline visit | Numerical continuous |
| Home type | Self-report of flat/apartment, sheltered accommodation, house, bungalow; considered as: communal living space vs non-communal (house/bungalow) | Binary categorical |
| Household size | Self-report of number of people in household at baseline | Numerical discrete |
| Generations in household | Self-report of relationship to people in household. | Numerical discrete |
| Family/friends visit | Self-reported answer to the question “"How often do you visit friends or family or have them visit you?" Coded as 1 if once a week or more, 0 if less than once per week. | Binary categorical |
| Regular leisure activity | Self-report of at least weekly leisure activity outside the home including sports, pub, religious group, adult education classes; considered as: regular leisure activity vs no regular leisure activity | Binary categorical |
| Risk taking | Self-report answer to “tendency to take risks” at baseline visit: Yes/No. | Binary categorical |

**Supplementary Table 1 footnote:** BMI: body mass index; HES: Hospital Episode Statistics; MI: myocardial infarction; UKB: UK Biobank
