# Supplementary material for: Greater risk of severe COVID-19 in Black, Asian and Minority Ethnic populations is not explained by cardiometabolic, socioeconomic or behavioural factors, or by 25(OH)-vitamin D status: study of 1326 cases from the UK Biobank
Source: J Public Health (Oxf). 2020 Jun 19;42(3):451–60. doi: 10.1093/pubmed/fdaa095 (PMC7449237; doi:10.1093/pubmed/fdaa095)
Supplement: Supplementary_Table_2_fdaa095 [file supplementary_table_2_fdaa095.docx]

**Supplementary Table 2. International Classification of Disease (ICD) codes used to define comorbidities from Hospital Episode Statistic data**

| Condition | ICD code | Code description |
| --- | --- | --- |
| Diabetes | E100 | Type 1 diabetes mellitus: With coma |
| Diabetes | E101 | Type 1 diabetes mellitus: With ketoacidosis |
| Diabetes | E102 | Type 1 diabetes mellitus: With renal complications |
| Diabetes | E103 | Type 1 diabetes mellitus: With ophthalmic complications |
| Diabetes | E104 | Type 1 diabetes mellitus: With neurological complications |
| Diabetes | E105 | Type 1 diabetes mellitus: With peripheral circulatory complications |
| Diabetes | E106 | Type 1 diabetes mellitus: With other specified complications |
| Diabetes | E107 | Type 1 diabetes mellitus: With multiple complications |
| Diabetes | E108 | Type 1 diabetes mellitus: With unspecified complications |
| Diabetes | E109 | Type 1 diabetes mellitus: Without complications |
| Diabetes | E110 | Type 2 diabetes mellitus: With coma |
| Diabetes | E111 | Type 2 diabetes mellitus: With ketoacidosis |
| Diabetes | E112 | Type 2 diabetes mellitus: With renal complications |
| Diabetes | E113 | Type 2 diabetes mellitus: With ophthalmic complications |
| Diabetes | E114 | Type 2 diabetes mellitus: With neurological complications |
| Diabetes | E115 | Type 2 diabetes mellitus: With peripheral circulatory complications |
| Diabetes | E116 | Type 2 diabetes mellitus: With other specified complications |
| Diabetes | E117 | Type 2 diabetes mellitus: With multiple complications |
| Diabetes | E118 | Type 2 diabetes mellitus: With unspecified complications |
| Diabetes | E119 | Type 2 diabetes mellitus: Without complications |
| Diabetes | E130 | Other specified diabetes mellitus: With coma |
| Diabetes | E131 | Other specified diabetes mellitus: With ketoacidosis |
| Diabetes | E132 | Other specified diabetes mellitus: With renal complications |
| Diabetes | E133 | Other specified diabetes mellitus: With ophthalmic complications |
| Diabetes | E134 | Other specified diabetes mellitus: With neurological complications |
| Diabetes | E135 | Other specified diabetes mellitus: With peripheral circulatory complications |
| Diabetes | E136 | Other specified diabetes mellitus: With other specified complications |
| Diabetes | E137 | Other specified diabetes mellitus: With multiple complications |
| Diabetes | E138 | Other specified diabetes mellitus: With unspecified complications |
| Diabetes | E139 | Other specified diabetes mellitus: Without complications |
| Diabetes | E140 | Unspecified diabetes mellitus: With coma |
| Diabetes | E141 | Unspecified diabetes mellitus: With ketoacidosis |
| Diabetes | E142 | Unspecified diabetes mellitus: With renal complications |
| Diabetes | E143 | Unspecified diabetes mellitus: With ophthalmic complications |
| Diabetes | E144 | Unspecified diabetes mellitus: With neurological complications |
| Diabetes | E145 | Unspecified diabetes mellitus: With peripheral circulatory complications |
| Diabetes | E146 | Unspecified diabetes mellitus: With other specified complications |
| Diabetes | E147 | Unspecified diabetes mellitus: With multiple complications |
| Diabetes | E148 | Unspecified diabetes mellitus: With unspecified complications |
| Diabetes | E149 | Unspecified diabetes mellitus: Without complications |
| Diabetes | G590 | Diabetic mononeuropathy |
| Diabetes | G632 | Diabetic polyneuropathy |
| Diabetes | H280 | Diabetic cataract |
| Diabetes | H360 | Diabetic retinopathy |
| Diabetes | M142 | Diabetic arthropathy |
| Diabetes | N083 | Glomerular disorders in diabetes mellitus |
| Diabetes | O240 | Diabetes mellitus in pregnancy: Pre-existing type 1 diabetes mellitus |
| Diabetes | O241 | Diabetes mellitus in pregnancy: Pre-existing type 2 diabetes mellitus |
| Diabetes | O243 | Diabetes mellitus in pregnancy: Pre-existing diabetes mellitus, unspecified |
| Diabetes | O244 | Diabetes mellitus arising in pregnancy |
| Diabetes | O249 | Diabetes mellitus in pregnancy, unspecified |
| Diabetes | Y423 | Insulin and oral hypoglycaemic [antidiabetic] drugs |
| Hypertension | I10X | Essential (primary) hypertension |
| Hypertension | I110 | Hypertensive heart disease with (congestive) heart failure |
| Hypertension | I119 | Hypertensive heart disease without (congestive) heart failure |
| Hypertension | I120 | Hypertensive renal disease with renal failure |
| Hypertension | I129 | Hypertensive renal disease without renal failure |
| Hypertension | I130 | Hypertensive heart and renal disease with (congestive) heart failure |
| Hypertension | I131 | Hypertensive heart and renal disease with renal failure |
| Hypertension | I132 | Hypertensive heart and renal disease with both (congestive) heart failure and renal failure |
| Hypertension | I139 | Hypertensive heart and renal disease, unspecified |
| High cholesterol | E780 | Pure hypercholesterolaemia |
| High cholesterol | E782 | Mixed hyperlipidaemia |
| High cholesterol | E783 | Hyperchylomicronaemia |
| High cholesterol | E784 | Other hyperlipidaemia |
| High cholesterol | E785 | Hyperlipidaemia, unspecified |
