# Supplementary material for: Greater risk of severe COVID-19 in Black, Asian and Minority Ethnic populations is not explained by cardiometabolic, socioeconomic or behavioural factors, or by 25(OH)-vitamin D status: study of 1326 cases from the UK Biobank
Source: J Public Health (Oxf). 2020 Jun 19;42(3):451–60. doi: 10.1093/pubmed/fdaa095 (PMC7449237; doi:10.1093/pubmed/fdaa095)
Supplement: Supplementary_Table_3_fdaa095 [file supplementary_table_3_fdaa095.docx]

**Supplementary Table 3. Baseline demographics by COVID-19 status**

|  | Test positive (*n*=1,326) | Test negative (*n*=3,184) | Untested (*n*=497,996) |
| --- | --- | --- | --- |
| Men | 696 (52.5%) | 1,505 (47.3%) | 226,921 (45.6%) |
| Age | 68.11 (± 9.23) | 68.91 (± 8.72) | 68.25 (± 8.10) |
| White ethnicity | 1,141 (86.0%) | 2,927 (91.9%) | 468,629 (94.1%) |
| Non-White ethnicity | 174 (13.1%) | 241 (7.6%) | 26,618 (5.3%) |
| Black ethnicity | 76 (5.7%) | 91 (2.9%) | 7,894 (1.6%) |
| Asian ethnicity | 60 (4.5%) | 78 (2.4%) | 9,744 (2.0%) |
| Chinese ethnicity | 6 (0.5%) | 3 (0.1%) | 1,565 (0.3%) |
| Mixed ethnicity | 9 (0.7%) | 24 (0.8%) | 2,925 (0.6%) |
| Other ethnicity* | 34 (2.6%) | 61 (1.9%) | 7,239 (1.5%) |
| Smoking (current or previous) | 683 (51.5%) | 1,653 (51.9%) | 225,902 (45.4%) |
| Processed meat intake (g/day) | 17.08 (± 15.67) | 16.33 (± 15.00) | 15.91 (± 14.94) |
| BMI (kg/m^2^) | 28.04 [± 6.47] | 27.41 [± 6.37] | 26.74 [± 5.77] |
| Diabetes | 217 (16.4%) | 449 (14.1%) | 38,472 (7.7%) |
| Hypertension | 624 (47.1%) | 1,457 (45.8%) | 172,913 (34.7%) |
| High cholesterol | 437 (33.0%) | 1,034 (32.5%) | 116,225 (23.3%) |
| Prior myocardial infarction | 96 (7.2%) | 242 (7.6%) | 20,477 (4.1%) |
| Vitamin D (nmol/L)** | 33.88 [± 27.01] | 35.45 [± 26.78] | 37.55 [± 26.49] |
| Townsend deprivation score | -0.91 [± 5.34] | -1.55 [± 5.00] | -2.14 [± 4.19] |
| Home type (flat/apartment) | 191 (14.4%) | 455 (14.3%) | 51,087 (10.3%) |
| Household size | 2.50 (± 1.31) | 2.32 (± 1.22) | 2.39 (± 1.15) |
| Number of generations in household | 1.41 (± 0.52) | 1.35 (± 0.50) | 1.37 (± 0.50) |
| Family/friend visits | 975 (73.5%) | 2,438 (76.6%) | 384,280 (77.2%) |
| Regular leisure activity | 897 (67.6%) | 2,124 (66.7%) | 344,518 (69.2%) |
| Tendency to take risks | 404 (30.5%) | 916 (28.8%) | 127,913 (25.7%) |

**Supplementary Table 3 footnote:** Results are number (percentage) for categorical and mean (standard deviation) or median [interquartile range] for continuous variables. **Ethnicity was missing for <1% of participants across all categories; they are displayed as part of “other ethnicity” in this table but have been excluded from subsequent modelling. *Vitamin D has been adjusted for seasonality.
