# Supplementary material for: Greater risk of severe COVID-19 in Black, Asian and Minority Ethnic populations is not explained by cardiometabolic, socioeconomic or behavioural factors, or by 25(OH)-vitamin D status: study of 1326 cases from the UK Biobank
Source: J Public Health (Oxf). 2020 Jun 19;42(3):451–60. doi: 10.1093/pubmed/fdaa095 (PMC7449237; doi:10.1093/pubmed/fdaa095)
Supplement: Supplementary_Table_4_fdaa095 [file supplementary_table_4_fdaa095.docx]

**Supplementary Table 4. Baseline characteristics stratified by sex and COVID-19 status**

|  | Test positive (*n*=1,326) | | Test negative (*n*=3,184) | | Untested (*n*=497,996) | | |
| --- | --- | --- | --- | --- | --- | --- | --- |
|  | Men (n=696) | Women (n=630) | Men (n=1,505) | Women (n=1,679) | Men (n=226,921) | Women (n=271,075) |  |
| Age | 69.65 (± 8.83) | 66.41 (± 9.36) | 70.24 (± 8.30) | 67.72 (± 8.92) | 68.46 (± 8.20) | 68.08 (± 8.01) |  |
| White ethnicity | 600 (86.2%) | 541 (85.9%) | 1,397 (92.8%) | 1,530 (91.1%) | 213,262 (94.0%) | 255,367 (94.2%) |  |
| Non-White ethnicity | 89 (12.8%) | 85 (13.5%) | 99 (6.6%) | 142 (8.5%) | 12,164 (5.4%) | 14,454 (5.3%) |  |
| Black ethnicity | 38 (5.5%) | 38 (6.0%) | 28 (1.9%) | 63 (3.8%) | 3,341 (1.5%) | 4,553 (1.7%) |  |
| Asian ethnicity | 32 (4.6%) | 28 (4.4%) | 46 (3.1%) | 32 (1.9%) | 5,216 (2.3%) | 4,528 (1.7%) |  |
| Chinese ethnicity | 1 (0.1%) | 5 (0.8%) | 0 (0.0%) | 3 (0.2%) | 583 (0.3%) | 982 (0.4%) |  |
| Mixed ethnicity | 5 (0.7%) | 4 (0.6%) | 7 (0.5%) | 17 (1.0%) | 1,093 (0.5%) | 1,832 (0.7%) |  |
| Other ethnicity* | 20 (2.9%) | 14 (2.2%) | 27 (1.8%) | 34 (2.0%) | 3,426 (1.5%) | 3,813 (1.4%) |  |
| Smoking (current, previous) | 422 (60.6%) | 261 (41.4%) | 887 (58.9%) | 766 (45.6%) | 115,977 (51.1%) | 109,925 (40.6%) |  |
| Processed meat intake (g/day) | 20.43 (± 16.46) | 13.38 (± 13.86) | 20.74 (± 16.21) | 12.39 (± 12.60) | 20.24 (± 16.32) | 12.29 (± 12.58) |  |
| BMI (kg/m^2^) | 28.31 [± 5.55] | 27.61 [± 7.43] | 27.77 [± 5.60] | 27.04 [± 7.12] | 27.30 [± 5.08] | 26.12 [± 6.27] |  |
| Diabetes | 140 (20.1%) | 77 (12.2%) | 264 (17.5%) | 185 (11.0%) | 22,829 (10.1%) | 15,643 (5.8%) |  |
| Hypertension | 380 (54.6%) | 244 (38.7%) | 824 (54.8%) | 633 (37.7%) | 90,879 (40.0%) | 82,034 (30.3%) |  |
| High cholesterol | 293 (42.1%) | 144 (22.9%) | 621 (41.3%) | 413 (24.6%) | 68,010 (30.0%) | 48,215 (17.8%) |  |
| Prior MI | 76 (10.9%) | 20 (3.2%) | 182 (12.1%) | 60 (3.6%) | 15,521 (6.8%) | 4,956 (1.8%) |  |
| Vitamin D** | 34.80 [± 28.32] | 33.07 [± 25.63] | 36.19 [± 26.80] | 34.58 [± 26.54] | 37.62 [± 25.81] | 37.49 [± 27.10] |  |
| Townsend deprivation score | -0.98 [± 5.51] | -0.90 [± 5.15] | -1.64 [± 5.12] | -1.41 [± 4.84] | -2.13 [± 4.27] | -2.15 [± 4.11] |  |
| House (Flat/Apartment) | 119 (17.1%) | 72 (11.4%) | 244 (16.2%) | 211 (12.6%) | 24,842 (10.9%) | 26,245 (9.7%) |  |
| Household size | 2.46 (± 1.34) | 2.54 (± 1.28) | 2.30 (± 1.17) | 2.34 (± 1.26) | 2.45 (± 1.17) | 2.34 (± 1.13) |  |
| Generations in household | 1.36 (± 0.51) | 1.47 (± 0.53) | 1.31 (± 0.48) | 1.39 (± 0.51) | 1.36 (± 0.50) | 1.37 (± 0.51) |  |
| Family/friend visits | 483 (69.4%) | 492 (78.1%) | 1,092 (72.6%) | 1,346 (80.2%) | 164,568 (72.5%) | 219,712 (81.1%) |  |
| Leisure activity | 485 (69.7%) | 412 (65.4%) | 1,006 (66.8%) | 1,118 (66.6%) | 157,461 (69.4%) | 187,057 (69.0%) |  |
| Tendency to take risks | 245 (35.2%) | 159 (25.2%) | 547 (36.3%) | 369 (22.0%) | 75,866 (33.4%) | 52,047 (19.2%) |  |

**Supplementary Table 4 footnote:** BMI: body mass index; COVID-19: coronavirus disease 2019; MI: myocardial infarction**Ethnicity was missing for <1% of participants across all categories; they are displayed as part of “other ethnicity” in this table but have been excluded from subsequent modelling. *Vitamin D has been adjusted for seasonality.
