# Supplementary material for: Greater risk of severe COVID-19 in Black, Asian and Minority Ethnic populations is not explained by cardiometabolic, socioeconomic or behavioural factors, or by 25(OH)-vitamin D status: study of 1326 cases from the UK Biobank
Source: J Public Health (Oxf). 2020 Jun 19;42(3):451–60. doi: 10.1093/pubmed/fdaa095 (PMC7449237; doi:10.1093/pubmed/fdaa095)
Supplement: Supplementary_Table_5_fdaa095 [file supplementary_table_5_fdaa095.docx]

**Supplementary Table 5. Baseline characteristics stratified by ethnicity and COVID-19 status**

|  | Test positive (*n*=1,326) | | Test negative (*n*=3,184) | | Untested (*n*=497,996) | |
| --- | --- | --- | --- | --- | --- | --- |
|  | White (n=1,141) | Non-White (n=174) | White (n=2,927) | Non-White (n=241) | White (n=468,629) | Non-White (n=26,618) |
| Men | 600 (52.6%) | 89 (51.1%) | 1,397 (47.7%) | 99 (41.1%) | 213,262 (45.5%) | 12,164 (45.7%) |
| Age | 68.75 (± 9.21) | 63.80 (± 8.16) | 69.27 (± 8.62) | 64.45 (± 8.86) | 68.50 (± 8.03) | 63.95 (± 8.18) |
| Black ethnicity |  | 76 (43.7%) |  | 91 (37.8%) |  | 7,894 (29.7%) |
| Asian ethnicity |  | 60 (34.5%) |  | 78 (32.4%) |  | 9,744 (36.6%) |
| Chinese ethnicity |  | 6 (3.4%) |  | 3 (1.2%) |  | 1,565 (5.9%) |
| Mixed ethnicity |  | 9 (5.2%) |  | 24 (10.0%) |  | 2,925 (11.0%) |
| Other ethnicity |  | 23 (13.2%) |  | 45 (18.7%) |  | 4,490 (16.9%) |
| Smoking (current, previous) | 621 (54.4%) | 56 (32.2%) | 1,566 (53.5%) | 74 (30.7%) | 215,755 (46.0%) | 8,298 (31.2%) |
| Processed meat intake (g/day) | 17.56 (± 15.47) | 14.04 (± 16.79) | 16.67 (± 14.97) | 12.35 (± 14.91) | 16.11 (± 14.91) | 12.38 (± 15.00) |
| BMI (kg/m^2^) | 27.88 [± 6.33] | 29.03 [± 7.99] | 27.38 [± 6.33] | 27.85 [± 7.11] | 26.71 [± 5.74] | 27.08 [± 6.01] |
| Diabetes | 176 (15.4%) | 37 (21.3%) | 380 (13.0%) | 66 (27.4%) | 33,587 (7.2%) | 4,518 (17.0%) |
| Hypertension | 523 (45.8%) | 94 (54.0%) | 1,331 (45.5%) | 116 (48.1%) | 161,763 (34.5%) | 10,170 (38.2%) |
| High cholesterol | 377 (33.0%) | 56 (32.2%) | 947 (32.4%) | 82 (34.0%) | 108,513 (23.2%) | 7,044 (26.5%) |
| Prior MI | 83 (7.3%) | 11 (6.3%) | 226 (7.7%) | 13 (5.4%) | 19,217 (4.1%) | 1,095 (4.1%) |
| Vitamin D | 35.60 [± 26.88] | 22.53 [± 18.70] | 36.29 [± 27.19] | 25.77 [± 20.58] | 38.28 [± 26.35] | 25.18 [± 21.14] |
| Townsend deprivation score | -1.31 [± 4.86] | 2.56 [± 5.49] | -1.76 [± 4.64] | 1.79 [± 6.15] | -2.26 [± 3.96] | 1.03 [± 5.61] |
| House (Flat/Apartment) | 140 (12.3%) | 49 (28.2%) | 379 (12.9%) | 73 (30.3%) | 44,241 (9.4%) | 6,505 (24.4%) |
| Household size | 2.39 (± 1.24) | 3.20 (± 1.57) | 2.26 (± 1.16) | 3.07 (± 1.66) | 2.35 (± 1.10) | 3.08 (± 1.63) |
| Generations in household | 1.37 (± 0.51) | 1.68 (± 0.54) | 1.33 (± 0.48) | 1.67 (± 0.58) | 1.35 (± 0.50) | 1.62 (± 0.57) |
| Family/friend visits | 868 (76.1%) | 101 (58.0%) | 2,276 (77.8%) | 156 (64.7%) | 366,439 (78.2%) | 16,684 (62.7%) |
| Leisure activity | 772 (67.7%) | 123 (70.7%) | 1,950 (66.6%) | 168 (69.7%) | 325,635 (69.5%) | 17,761 (66.7%) |
| Tendency to take risks | 334 (29.3%) | 69 (39.7%) | 822 (28.1%) | 91 (37.8%) | 117,718 (25.1%) | 9,635 (36.2%) |

**Supplementary Table 5 footnote:** BMI: body mass index; COVID-19: coronavirus disease 2019; MI: myocardial infarction
