# Supplementary material for: Greater risk of severe COVID-19 in Black, Asian and Minority Ethnic populations is not explained by cardiometabolic, socioeconomic or behavioural factors, or by 25(OH)-vitamin D status: study of 1326 cases from the UK Biobank
Source: J Public Health (Oxf). 2020 Jun 19;42(3):451–60. doi: 10.1093/pubmed/fdaa095 (PMC7449237; doi:10.1093/pubmed/fdaa095)
Supplement: Supplementary_Table_6_fdaa095 [file supplementary_table_6_fdaa095.docx]

**Supplementary Table 6. Univariate logistic regression models exposures associations with COVID-19 status in whole cohort, men, and women within the tested sample (n=4,510)**

| **Exposures** | **Whole sample** | **Men** | **Women** |
| --- | --- | --- | --- |
|  | Test positive, *n*=1,326  Test negative, *n*=3,184 | Test positive, *n*=696  Test negative, *n*=1,505 | Test positive, *n*=630  Test negative, *n*=1,679 |
| Sex (Male) | 1.23* [1.08, 1.40] | – | – |
|  | 0.0014 | – | – |
| Age | 0.99* [0.98, 1.00] | 0.99 [0.98, 1.00] | 0.98* [0.97, 0.99] |
|  | 0.0059 | 0.1290 | 0.0020 |
| Ethnicity (Non-white) | 1.85* [1.51, 2.28] | 2.09* [1.55, 2.83] | 1.69* [1.27, 2.25] |
|  | 5.03$\times$10^-9^ | 1.62$\times$10^-06^ | 3.11$\times$10^-04^ |
| Townsend deprivation score | 1.04* [1.02, 1.06] | 1.04* [1.02, 1.07] | 1.05* [1.02, 1.07] |
|  | 6.92$\times$10^-6^ | 0.0015 | 0.0017 |
| Home Type (flat/apartment) | 1.01 [0.84, 1.21] | 1.07 [0.84, 1.36] | 0.892 [0.667, 1.180] |
|  | 0.9439 | 0.5936 | 0.4294 |
| Household Size | 1.12* [1.06, 1.17] | 1.11* [1.03, 1.20] | 1.12* [1.05, 1.21] |
|  | 1.80$\times$10^-5^ | 0.0040 | 0.0011 |
| Generations in household | 1.26* [1.11, 1.43] | 1.21* [1.01, 1.45] | 1.35* [1.14, 1.61] |
|  | 3.32$\times$10^-4^ | 0.0374 | 7.46$\times$10^-04^ |
| Family/friend visits | 0.84* [0.72, 0.98] | 0.85 [0.70, 1.04] | 0.87 [0.69, 1.11] |
|  | 0.0264 | 0.1184 | 0.2584 |
| Socialisation habits | 1.04 [0.91, 1.19] | 1.14 [0.94, 1.39] | 0.94 [0.77, 1.14] |
|  | 0.5848 | 0.1864 | 0.5269 |
| Processed meat intake | 1.38 [0.90, 2.09] | 0.89 [0.51, 1.55] | 1.78 [0.88, 3.53] |
|  | 0.1354 | 0.6767 | 0.1036 |
| Diabetes | 1.19 [1.00, 1.42] | 1.18 [0.94, 1.49] | 1.12 [0.84, 1.49] |
|  | 0.0512 | 0.1473 | 0.4168 |
| Hypertension | 1.05 [0.93, 1.20] | 0.99 [0.83, 1.19] | 1.05 [0.87, 1.26] |
|  | 0.4254 | 0.9465 | 0.6499 |
| High Cholesterol | 1.02 [0.89, 1.17] | 1.04 [0.86, 1.24] | 0.91 [0.73, 1.13] |
|  | 0.7534 | 0.7116 | 0.3839 |
| Body mass index (kg/m^2^) | 1.02* [1.01, 1.04] | 1.03* [1.01, 1.05] | 1.02* [1.00, 1.03] |
|  | 8.07$\times$10^-05^ | 0.0010 | 0.0221 |
| Smoking (current/previous) | 0.98 [0.87, 1.12] | 1.07 [0.89, 1.29] | 0.84 [0.70, 1.01] |
|  | 0.8029 | 0.4513 | 0.0710 |
| Prior myocardial infarction | 0.95 [0.74, 1.21] | 0.89 [0.67, 1.18] | 0.89 [0.52, 1.45] |
|  | 0.6752 | 0.4263 | 0.6408 |
| Vitamin D | 1.00 [0.99, 1.00] | 1.00 [0.99, 1.00] | 1.00 [0.99, 1.00] |
|  | 0.6386 | 0.6256 | 0.7117 |
| Risk Taking | 1.09 [0.94, 1.25] | 0.95 [0.79, 1.15] | 1.20 [0.97, 1.48] |
|  | 0.2534 | 0.6030 | 0.0968 |

**Supplementary Table 6 footnote:** COVID-19: coronavirus disease 2019
