# Supplementary material for: Greater risk of severe COVID-19 in Black, Asian and Minority Ethnic populations is not explained by cardiometabolic, socioeconomic or behavioural factors, or by 25(OH)-vitamin D status: study of 1326 cases from the UK Biobank
Source: J Public Health (Oxf). 2020 Jun 19;42(3):451–60. doi: 10.1093/pubmed/fdaa095 (PMC7449237; doi:10.1093/pubmed/fdaa095)
Supplement: Supplementary_Table_7_fdaa095 [file supplementary_table_7_fdaa095.docx]

**Supplementary Table 7. Multivariate logistic regression models testing the role of socialisation habits (Model A), and attitude to risk (Model B) in determining risk of COVID-19**

|  | Exposures | Whole tested sample  *n=*4,510 | Men  *n=*2,201 | Women  *n=*2,309 |
| --- | --- | --- | --- | --- |
| *Model A: age, sex, ethnicity, socialisation habits* | Male sex | 1.27* [1.11, 1.46] | – | – |
|  |  | 4.78$\times$10^-4^ | – | – |
|  | Age | 0.99* [0.98, 1.00] | 1.00 [0.99, 1.01] | 0.99* [0.98, 1.00] |
|  |  | 0.0129 | 0.4313 | 0.0099 |
|  | Non-White ethnicity | 1.77* [1.43, 2.20] | 2.12* [1.54, 2.91] | 1.53* [1.13, 2.06] |
|  |  | 2.03$\times$10^-7^ | 3.31$\times$10^-6^ | 0.0050 |
|  | Family/friend visits | 0.91 [0.78, 1.06] | 0.88 [0.71, 1.08] | 0.95 [0.75, 1.21] |
|  |  | 0.2340 | 0.2073 | 0.6886 |
|  | Regular leisure activity | 1.05 [0.91, 1.21] | 1.15 [0.94, 1.41] | 0.95 [0.78, 1.16] |
|  |  | 0.5104 | 0.1633 | 0.6149 |
| *Model B: age, sex, ethnicity, risk taking* | Male sex | 1.27* [1.11, 1.45] | – | – |
|  |  | 4.07$\times$10^-4^ | – | – |
|  | Age | 0.99* [0.98, 1.00] | 1.00 [0.99, 1.01] | 0.99* [0.98, 1.00] |
|  |  | 0.0191 | 0.5423 | 0.0114 |
|  | Non-White ethnicity | 1.79* [1.45, 2.21] | 2.06* [1.51, 2.82] | 1.60* [1.19, 2.13] |
|  |  | 5.99$\times$10^-8^ | 4.71$\times$10^-6^ | 0.0016 |
|  | Risk taking | 1.02 [0.88, 1.17] | 0.93 [0.77, 1.12] | 1.14 [0.92, 1.42] |
|  |  | 0.8377 | 0.4340 | 0.2221 |

**Supplementary Table 7 footnote:** Results are odds ratios, 95% confidence interval, and p-values for each exposure from two separate models (A, B). Exposures are mutually adjusted.
